# Assessment of eye fatigue caused by head-mounted displays using eye-tracking

**DOI:** 10.1186/s12938-019-0731-5

**Published:** 2019-11-15

**Authors:** Yan Wang, Guangtao Zhai, Sichao Chen, Xiongkuo Min, Zhongpai Gao, Xuefei Song

**Affiliations:** 1 Institute of Image Communication and Network Engineering, Shanghai Key Laboratory of Digital Media Processing and Transmissions, Shanghai Jiao Tong University, Shanghai, China; 2 Institute of Ophthalmology and Vision Science of Beijing, Beijing, China; 3 Department of Ophthalmology, Ninth People’s Hospital, Shanghai Jiao Tong University School of Medicine, Shanghai, China; 4 Shanghai Key Laboratory of Orbital Diseases and Ocular Oncology, Shanghai, China; 5 Clinical Research Center, Shanghai Jiao Tong University School of Medicine, Shanghai, China

**Keywords:** Eye fatigue, Head-mounted display, Accommodation response, Eye movement

## Abstract

**Background:**

Head-mounted displays (HMDs) and virtual reality (VR) have been frequently used in recent years, and a user’s experience and computation efficiency could be assessed by mounting eye-trackers. However, in addition to visually induced motion sickness (VIMS), eye fatigue has increasingly emerged during and after the viewing experience, highlighting the necessity of quantitatively assessment of the detrimental effects. As no measurement method for the eye fatigue caused by HMDs has been widely accepted, we detected parameters related to optometry test. We proposed a novel computational approach for estimation of eye fatigue by providing various verifiable models.

**Results:**

We implemented three classifications and two regressions to investigate different feature sets, which led to present two valid assessment models for eye fatigue by employing blinking features and eye movement features with the ground truth of indicators for optometry test. Three graded results and one continuous result were provided by each model, respectively, which caused the whole result to be repeatable and comparable.

**Conclusion:**

We showed differences between VIMS and eye fatigue, and we also presented a new scheme to assess eye fatigue of HMDs users by analysis of parameters of the eye tracker.

## Background

Application of virtual reality (VR) with head-mounted displays (HMDs) possesses great advantages for the evaluation and therapy of clinical disorders. Among health problems, eye fatigue and visually induced motion sickness (VIMS) are highly concerned [1–4]. These display technologies possess a variety of advantages compared with traditional systems, while a remarkable stress is imposed due to the existence of a shorter distance from screen to observer’s eyes. Several psychophysiological and behavioral methods were utilized to indicate how VIMS affects electrodermal activity for a single user within a controlled environment [5, 6]. However, lack of a widely accepted measurement method for eye fatigue is highly tangible.

Symptoms of eye fatigue can be identified through subjective feelings and objective indicators. Subjective feelings include various kinds of symptoms, such as soreness of the eyes, headache, and tiredness [7]. Ohno and Ukai [8] found that subjective feelings on eye fatigue included descriptions, such as “trouble focusing,” “hazy,” “gritty,” “near-vision difficulty,” and “far-vision difficulty.” Hockey et al. [9] defined eye fatigue as a decrease in the performance of a particular mission. The objective indicators involve critical fusion frequency (CFF) [10], binocular vision [11], eye-blink rate (EBR) [12], and pupil constriction rate [13]. Changes in accommodative and vergence functions were reported to occur after working periods at a visual display terminal (VDT), and these changes were proposed as objective indicators for visual fatigue [14].

Bando et al. demonstrated that the difference between natural observation of real-world scenes versus display technology may cause visual discomfort and eye fatigue [15]. Eye fatigue in HMDs is mainly caused by the vergence–accommodation conflict [16]. This kind of conflict is created by a mismatch between perceived and virtual depth. In order to obtain a clear vision, our eyes converge and accommodate by a level dependent on the distance to the viewing object. When eyes converge to look at the object, accommodation changes the eye’s lens to obtain and maintain the object in the fovea. When the HMD is used in a stereoscopic mode, vergence is adjusted according to the virtual distance of the fixated object. However, accommodation is fixed in the HMD, and this is a cause of eye fatigue because accommodation and vergence are cross-linked functions. Fatigue has been mainly defined in association with muscle performance [17]. In the eye muscles, the same case can be assumed since eye movement is habitual from birth, eliminating symptoms of muscle fatigue [18]. In the present study, eye fatigue is defined as the functional decline in a number of specific eye muscles. Since extraocular muscles (EOMs) have been reported as fatigue-resistant muscles in the literature [19, 20] and EOMs can control different rotation movements of the eye [21, 22], the specific eye muscles are only among those control the activities inside the eyeball. We selected the muscles that control accommodation response, pupil size, and lens thickness as the specific muscles. Ciliary muscles control accommodation response and lens thickness [23]. The size of pupil can be controlled by the pupillary muscle plant which includes two kinds of antagonistic muscles: the sphincter and dilator [24]. We demonstrated that these muscles have a short-term stable functional decline when eye fatigue occurred. Even though this definition can be used as a measurement of eye fatigue, the measurement process is complex and non-real time.

Questionnaires were developed to subjectively assess visual fatigue caused by observation of various types of visual stimuli. The questionnaires were evaluated using four types of moving images: playing a TV game using an HMD or a TV, viewing images with and without elaborating camera shake, viewing a movie with and without color breakup, and viewing either a stereoscopic movie (anaglyph method) or a non-stereoscopic movie [7, 25]. Emoto et al. assessed and compared visual fatigue by measuring fusional amplitude [26]. Li et al. found that delay in transmission of visual information measured by electroencephalogram (EEG) was efficient in visual fatigue [27]. A number of scholars also attempted to assess eye fatigue by measuring brain activity; however, their approach was indeed complicated, as well as being very sensitive to the individual state of the subjects. Thus, a series of subjective indexes had been captured by optometry instruments and accommodation response were explored as indicators of visual fatigue [14, 28]. Moreover, eye movement was employed to assess mental fatigue and visual fatigue [29, 30]. It was revealed that EBR decreased in HMD environment compared with that in natural environment [30]. However, due to equipment limitations, EBR could not be measured when the subject was using the HMD. Some scholars measured EBR only in the natural environment for both experimental group and control group. To deeply increase the scientific knowledge on eye fatigue and eye activities, Kim et al. proposed a visual fatigue monitoring system based on eye movement and eye-blink detection [31]. They found that the saccade movement of the eye decreased, while the frequency of eye-blink increased when eye fatigue was accumulated due to the increase in fixation time. In addition, a new assessment of eye fatigue related to three-dimensional (3D) display was proposed based on multimodal measurements [32]. To our knowledge, no research has assessed eye fatigue induced by HMDs using eye-tracking methods, even if eye-trackers can be embedded into HMDs [33, 34].

In order to overcome the shortcomings of previously reported studies, we proposed an objective algorithm to estimate eye fatigue caused by an HMD using eye-tracking data. Based on our previous research, we adopted seven objective indicators as the ground truth of eye fatigue, which were all subjective optometry data, including binocular crossed cylinder (BCC) test, negative relative accommodation (NRA), positive relative accommodation (PRA), left pupil diameter (PL), right pupil diameter (PR), left lens thickness (LTL), and right lens thickness (LTR) [35–40]. Furthermore, we further developed the concept of eye fatigue by proposing a new assessment strategy due to measurable eye activities (e.g., accommodation, pupil change, lens change, and eye movement).

## Results

### SSQ results

**Fig. 1:**
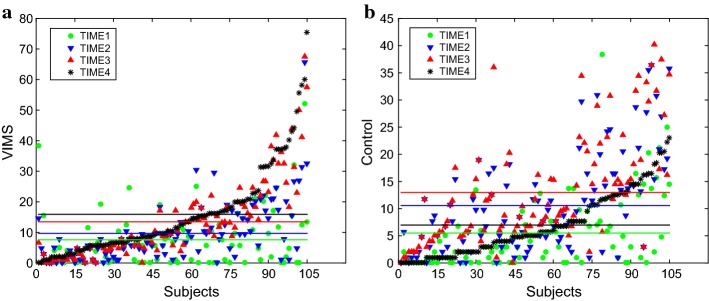
Changes of SSQ scores for the 105 subjects in four measurements, **a** main experiment, **b** control experiment

The SSQ scores of 105 subjects are presented in Fig. 1. Here, 105 subjects on the horizontal axis are arranged in ascending order of the data measured in the fourth SSQ. No consistent change can be observed through time of all the subjects, indicating that the subjects did not suffer from obvious VIMS after the experiment. For total of 420 samples, the linear correlations between SSQ and different indicators for optometry test are shown in Table 1. It can be seen that VIMS has a poor correlation with the feeling of “eye fatigue.” As all the subjects were fully relaxed before the experiment, all the 105 subjects were scored 0 in this item at the first SSQ, and it was revealed that only 41 out of 105 subjects were scored more than 0 in this item at the fourth SSQ.

**Table 1:** Linear correlation between SSQ and indicators of optometry test

	BCC (D)	NRA (D)	PRA (D)	PR (mm)	PL (mm)	LTR (mm)	LTL (mm)
SSQ	0.17	− 0.23	0.21	− 0.08	− 0.02	− 0.09	− 0.09

### Optometry results

Table 2 shows the mean values and the changes of the indicators in the fourth measurement of the main and control experiments. There was an increasing trend for *BCC*, while a decreasing trend was observed for pupil size, lens thickness, and the amplitude of *PRA* and *NRA* in the first measurement compared with the second, third, and fourth measurements; however, no obvious trend was found in the control experiment.

**Table 2:** Mean values and changes of optometry indicators in the main and control experiments

Experiment	Indicator	1st measurement	2nd measurement	3rd measurement	4th measurement
Main experiment	BCC(D)	0.14 ± 0.08	0.26 ± 0.34	0.36 ± 0.36	{0.50} ± {0.40}
	NRA (D)	2.35 ± 0.60	{2.15} ± {0.61}	{2.00} ± {0.62}	{1.79} ± {0.64}
	PRA (D)	− 2.74 ± 1.55	− 2.40 ± 1.50	− {2.11} ± {1.4}	− {1.78} ± {1.33}
	PR (mm)	5.37 ± 0.62	5.21 ± 0.60	{5.08} ± {0.6}	{4.93} ± {0.62}
	PL (mm)	5.29 ± 0.67	5.10 ± 0.63	{4.95} ± {0.65}	{4.79} ± {0.65}
	LTR (mm)	3.95 ± 0.36	3.90 ± 0.36	3.86 ± 0.36	{3.79} ± {0.36}
	LTL (mm)	3.95 ± 0.35	3.89 ± 0.35	3.86 ± 0.36	{3.77} ± {0.35}
Control experiment	BCC(D)	0.14 ± 0.28	0.14 ± 0.28	0.14 ± 0.28	0.14 ± 0.28
	NRA (D)	2.35 ± 0.60	2.35 ± 0.60	2.35 ± 0.60	2.35 ± 0.60
	PRA (D)	− 2.74 ± 1.55	− 2.74 ± 1.55	− 2.74 ± 1.55	− 2.74 ± 1.55
	PR (mm)	5.37 ± 0.62	5.37 ± 0.62	5.37 ± 0.61	5.37 ± 0.62
	PL (mm)	5.29 ± 0.67	5.29 ± 0.67	5.29 ± 0.66	5.29 ± 0.68
	LTR (mm)	3.95 ± 0.36	3.95 ± 0.36	3.94 ± 0.36	3.95 ± 0.36
	LTL (mm)	3.94 ± 0.35	3.95 ± 0.35	3.95 ± 0.35	3.95 ± 0.36

Values of indicators were shown as mean ± standard deviation (SD)

Compared with 1st measurement, $\hbox {P}<0.05$ were characterized in italics

### Weighted eye fatigue

**Fig. 2:**
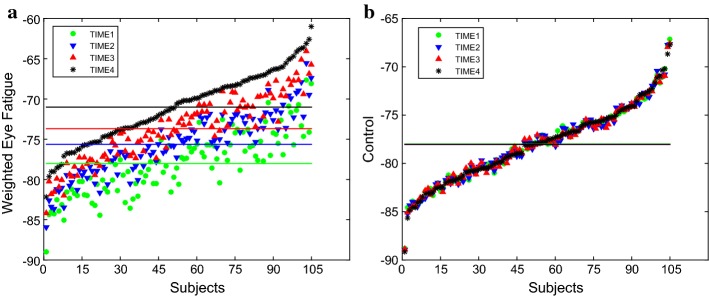
Weighted eye fatigue for the 105 subjects throughout the experiment, **a** main experiment, **b** control experiment

**Fig. 3:**
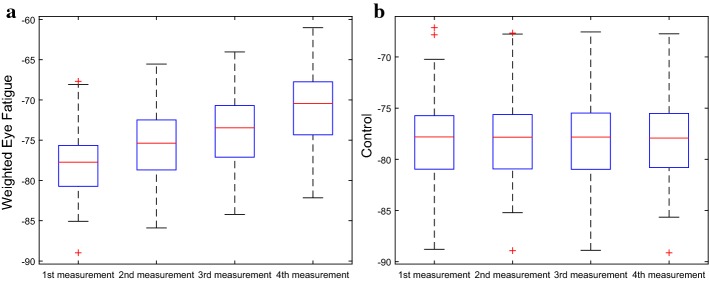
Changes of weighted eye fatigue throughout time, **a** main experiment, **b** control experiment

We proposed a weighted eye fatigue measurement based on seven indicators of optometry test. In order to demonstrate the reasonability of every indicator to eye fatigue, we conducted the Student’s *t* test on the seven indicators of optometry test. The results of statistical analysis are listed in Table 2. The significance level was set to 0.05. The results showed that all the indicators for optometry test showed significant differences in data collected from the beginning of the experiment to the end. Figure 2 shows the results of weighted eye fatigue of all the 105 subjects in the experiment computed by Eq. (4). The 105 subjects on the horizontal axis are arranged in ascending order of the data measured in the fourth time. Compared with healthy controls, the eye fatigue level of all the 105 subjects increased over time in the main experiment, which was caused by viewing the HMD display. The values of mean and standard deviation (SD) related to changes of the weighted fatigue are depicted in Fig. 3. The red crosses are outliers. It can be seen that in addition to mean values changes, the SD values of the subjects gradually changed with progress of the experiment.

**Table 3:** Mean values and changes of eye movement features in the main experiment

Eye movement features	1st measurement	2nd measurement	3rd measurement	4th measurement
Number of fixation points	$43.12\pm 7.97$	${ 39.50}\pm { 8.20}$	${ 35.62}\pm { 8.98}$	${ 30.19}\pm { 9.61}$
Total duration of fixation points (*s*)	$16.97\pm 0.56$	$16.88\pm 0.87$	${ 16.72}\pm { 1.08}$	${ 16.05}\pm { 1.75}$
Mean duration of fixation points (*s*)	$0.41\pm 0.12$	${ 0.46}\pm { 0.15}$	${ 0.53}\pm { 0.29}$	${ 0.67}\pm { 0.57}$
Variance of fixation durations ($s^{2}$)	$0.08\pm 0.06$	${ 0.13}\pm { 0.09}$	${ 0.40}\pm { 0.31}$	${ 0.91}\pm { 0.63}$
Times of blinking	$2.57\pm 2.44$	$4.65\pm 3.51$	${ 6.79}\pm { 4.50}$	${ 10.21}\pm { 6.18}$
Total duration of blinking (*s*)	$0.49\pm 0.49$	$0.95\pm 0.77$	${ 1.48}\pm { 1.05}$	${ 2.55}\pm { 1.73}$
Mean duration of blinking (*s*)	$0.16\pm 0.07$	$0.20\pm 0.03$	$0.21\pm 0.04$	$0.25\pm 0.07$
Variance of blink durations ($s^{2}$)	$4.01\hbox {E-}04\pm 3.05\hbox {E-}04$	${ 1.13E-03}\pm { 1.06E-03}$	${ 2.71E-03}\pm { 2.16E-03}$	${ 1.06E-02}\pm { 0.87E-02}$
Length of scanpath (*pixel*)	$6002.49\pm 3994.3$	${ 8387.62}\pm { 4824.81}$	${ 11109.34}\pm { 6576.00}$	${ 16063.95}\pm { 8713.61}$
Mean of saccade length (*pixel*)	$137.11\pm 84.5$	${ 209.91}\pm { 110.03}$	${ 331.59}\pm { 255.29}$	${ 619.30}\pm { 500.48}$

Values of indicators were shown as mean ± standard deviation (SD).

Compared with 1st measurement, $\hbox {P}<0.05$ were characterized in italics

Data were recorded during 20 s

### Eye movements results

Table 3 shows the values of mean and SD related to changes of eye movement features in the main experiment. There was an increasing trend for SD duration of fixation and mean duration of fixation, as well as a decreasing trend for the number of fixation and total duration of fixation in the 1st measurement compared with the 2nd, 3rd, and 4th measurements. The findings disclosed that the four blinking features were enhanced over time for all the participants, and their eye fatigue level increased as well. In general, individuals normally reduce EBR due to sleepiness or boredom. Compared with the 1st measurement, the 2nd, 3rd, and 4th measurements were increased, representing the increase in eye fatigue level. In the present study, Kolmogorov–Smirnov [41] test was used to assess whether data were normally distributed. In addition, the Student’s *t* test was carried out on all the ten features selected in this study, and the results are presented in Table 3. $\hbox {P}<0.05$ was considered statistically significant. It was revealed that there were significant differences in eye movement features between data collected from the beginning of the experiment to the end.

### Ranking the eye movement features

With the help of minimal-redundancy-maximal-relevance (MRMR) criterion [42], we ranked the ten eye movement features. The ranked features according to the unified weighted eye fatigue level are listed in Table 4. The total duration of computation was 37 s. The findings indicated that the top five features of the all three kinds of classifications were consistent.

**Table 4:** Ranking the eye movement features based on MRMR

Feature rank	Two-class classification	Three-class classification	Four-class classification
1	Length of scanpath	Length of scanpath	Length of scanpath
2	Mean duration of blinking	Mean duration of blinking	Mean duration of blinking
3	Variance of blink durations	Variance of blink durations	Variance of blink durations
4	Mean duration of fixation	Mean duration of fixation	Mean duration of fixation
5	Variance of fixation durations	Variance of fixation durations	Variance of fixation durations
6	Total duration of blinking	Total duration of blinking	Mean of saccade length
7	Total duration of fixation	Total duration of fixation	Total duration of blinking
8	Number of fixation	Number of fixation	Total duration of fixation
9	Times of blinking	Times of blinking	Number of fixation
10	Mean of saccade length	Mean of saccade length	Times of blinking

### Assessment results

We established eleven feature sets for analyzing by support vector machine (SVM). The first ten feature sets were named as Set 1 to Set 10, and the name of the 11th feature set was blink set. Set 1 contained one eye movement feature, Set 2 contained two eye movement features, and so forth. We applied the selection method of four-class classification for the epsilon regression and nonlinear regression. We established blink set to investigate whether the blink features alone are qualified to assess individuals’ eye fatigue, considering that it is more economic to achieve blinking features than general eye movement features.

**Table 5:** Accuracy of the three kinds of classifications

Feature	Accuracy of two-class classification				Accuracy of three-class classification				Accuracy of four-class classification			
	Kernel 1	Kernel 2	*Kernel 3*	Kernel 4	*Kernel 1*	Kernel 2	Kernel 3	Kernel 4	Kernel 1	*Kernel 2*	Kernel 3	Kernel 4
Feature set 1	0.8323	0.8310	0.8419	0.8503	0.6953	0.6849	0.6855	0.6481	0.6527	0.6269	0.6249	0.6163
Feature set 2	0.8353	0.8358	0.8460	0.8518	0.7042	0.6936	0.6955	0.6585	0.6565	0.6572	0.6530	0.6356
Feature set 3	0.8406	0.8404	0.8511	0.8542	0.7134	0.7040	0.7053	0.6709	0.6639	0.6886	0.6659	0.6454
Feature set 4	0.8511	0.8498	0.8608	0.8604	0.7308	0.7217	0.7252	0.6956	0.6778	0.7015	0.6844	0.6647
Feature set 5	0.8556	0.8550	0.8678	0.8637	0.7401	0.7319	0.7355	0.6964	0.6841	0.7084	0.6938	0.6743
Feature set 6	0.8603	0.8608	0.8751	0.8685	0.7496	0.7394	0.7450	0.7076	0.6913	0.7169	0.7037	0.6751
Feature set 7	0.8649	0.8644	0.8805	0.8720	0.7589	0.7489	0.7542	0.7190	0.6989	0.7209	0.7044	0.6846
Feature set 8	0.8708	0.8695	0.8869	0.8755	0.7690	0.7575	0.7651	0.7315	0.7053	0.7394	0.7145	0.6932
Feature set 9	0.8754	0.8742	0.8934	0.8781	0.7885	0.7656	0.7775	0.7435	0.7114	0.7398	0.7239	0.7040
*Feature set 10*	0.8887	0.8780	*0.9079*	0.8836	*0.7947*	0.7750	0.7853	0.7535	0.7276	*0.7425*	0.7248	0.7137
*Blink set*	0.8632	0.8479	*0.8763*	0.8613	*0.7458*	0.7267	0.7416	0.6952	0.6874	*0.7225*	0.7104	0.6739
Mean	0.8580	0.8552	*0.8716*	0.8654	*0.7446*	0.7317	0.7378	0.7018	0.6870	*0.7059*	0.6912	0.6710

We analyzed kernels as follows: linear kernel, polynomial kernel, radial basis function kernel, and sigmoid kernel, corresponding to kernel 1 to kernel 4, respectively. All the five SVMs were trained in each round, which lasted for 48 min. Table 5 shows the results of the three kinds of classifications. It was unveiled that the set 10 had the highest accuracy of classification compared with the other feature sets. It also was revealed that the maximum accuracy of the two-class classification, three-class classification, and four-class classification was 0.9079, 0.7947, and 0.7425, respectively. As displayed in Table 5, the blink set had a performance that was slightly worse than the feature set 10. Regarding the kernel selection, kernel 3, kernel 1, and kernel 2 were the most appropriate kernels for the three types of classifications, respectively. Table 6 represents the correlations between the regression results and the ground truth of eye fatigue. The feature set 10 also showed the best performance compared with the other feature sets. Once comparisons were made between the two regressions, it was revealed that the epsilon regression outperformed.

According to the results of classification and regression, two assessment models were established: an eye tracker model and a blink detector model. The eye tracker model used the feature set 10 as input data. The blink detector model utilized the blink set as input data. Both models had four applications, including three classifications and one regression. The mentioned three classifications used radial basis function kernel, linear kernel, and polynomial kernel, respectively. The regression was the epsilon regression using polynomial kernel. The performances of the two assessment models are listed in Table 7.

**Table 6:** Correlation between the regression results and the ground truth of eye fatigue

Feature	Correlation with epsilon regression				Correlation with nonlinear regression			
	Kernel 1	*Kernel 2*	Kernel 3	Kernel 4	Kernel 1	*Kernel 2*	Kernel 3	Kernel 4
Feature set 1	0.6271	0.6989	0.6807	0.0237	0.6266	0.6712	0.6414	0.0663
Feature set 2	0.7364	0.7616	0.7456	0.4662	0.7449	0.7713	0.7369	0.4217
Feature set 3	0.7416	0.7707	0.7548	0.5674	0.7471	0.7779	0.7551	0.5401
Feature set 4	0.7536	0.7853	0.7557	0.6317	0.7547	0.7827	0.7631	0.5926
Feature set 5	0.7621	0.7886	0.7681	0.6593	0.7556	0.7851	0.7663	0.6182
Feature set 6	0.8062	0.8435	0.8122	0.6751	0.8063	0.8316	0.8197	0.6375
Feature set 7	0.8489	0.8514	0.8313	0.6925	0.8416	0.8383	0.8398	0.6597
Feature set 8	0.8563	0.8552	0.8363	0.6946	0.8452	0.8423	0.8469	0.6656
Feature set 9	0.8575	0.8675	0.8421	0.7087	0.8559	0.8583	0.8483	0.6735
*Feature set 10*	0.8652	*0.8737*	0.8489	0.7104	0.8568	0.8651	0.8532	0.6857
*Blink set*	0.7655	*0.7966*	0.7745	0.4624	0.7541	0.7746	0.7609	0.4731
Mean	0.7837	*0.8085*	0.7864	0.5720	0.7808	0.7999	0.7847	0.5485

**Table 7:** The performance of the two assessment models

Model	Input data	Accuracy of two-class classification	Accuracy of three-class classification	Accuracy of four-class classification	Correlation with regression
Eye tracker model	Feature set 10	0.9079	0.7947	0.7425	0.8737
Blink detector model	Blink set	0.8763	0.7458	0.7225	0.7966

## Discussion

In the present research, an objective algorithm was developed to estimate eye fatigue caused by an HMD using eye-tracking data. We used a weighted combination of seven indicators for optometry test as the ground truth of eye fatigue, which was found to be a novel definition based on our previous study. Based on SSQ and optometry data, we note that the relationship between VIMS and the feeling of eye fatigue was insignificant. For further study on HMDs related to eye fatigue, an unobtrusive eye tracker was mounted on the VR gear, which led to achieving ten features related to eye movement. With the help of MRMR criterion for ranking the features, three kinds of classifications and two kinds of regressions were resulted to evaluate the performance for different feature sets.

A series of methods have been proposed to assess eye fatigue; however, the eye fatigue caused by HMD has been rarely investigated yet. Previous studies were mainly concentrated on VIMS when discomfort caused by HMD was target [43, 44]. In the present study, we first demonstrated that eye fatigue and VIMS are different. We used SSQ to assess the subjects’ VIMS throughout the experiment. Figure 1 shows that the majority of the subjects did not feel any VIMS after the experiment. Figure 2 depicts that all the 105 subjects suffered from eye fatigue after the experiment, indicating that eye fatigue is a muscular disorder, and subjective feeling of eye fatigue may associate with long consciousness after the occurrence of eye fatigue. A number of scholars considered the subjective feeling as the ground truth of the eye fatigue [26], which was not accurate. In the present experiment, there was also an item “eye fatigue” in the SSQ, in which 41 out of 105 subjects reported that they felt eye fatigue after the experiment, reflecting that subjective feelings cannot accurately measure eye fatigue. Li et al. found that delay in the transmission of visual information measured with EEG could be helpful for visual fatigue [27]. However, eye fatigue is taken as an eye problem in lieu of a brain problem. In the current research, we used seven indicators for optometry test, including BCC, NRA, PRA, PL, PR, LTL, and LTR to define eye fatigue. Besides, BCC, NRA, PRA, LTL, and LTR reflected the accommodative ability of the ciliary muscles. Additionally, PL and PR referred to the accommodative ability of pupillary muscle plant. As shown in Table 2, all the seven indicators are sensitive to eye fatigue. It is noteworthy that LTR and LTL decreased from 3.95 mm at the first measurement to 3.79 and 3.77 mm at the fourth measurement. Researchers found that prior to presbyopia, the thickness of lens increased by about 42 to 72 μm per diopter of accommodation [45–47]. In every optometry test of our experiment, the order of measurement was BCC, PRA, NRA, pupil size, and lens thickness. Pupil size and lens thickness were simultaneously measured by the optical biometer. For each subject, his/her lens thickness was measured right after NRA test. When NRA test was performed, the subject was added positive diopter, which led to decreasing of his/her lens thickness. When testing of lens thickness was undertaken, the subject observed a light source with a fix distance, which required accommodation. The results of the present experiment showed that lens thickness of the subjects decreased over time because their accommodative ability decreased as eye fatigue occurred.

Since the measurement based on optometry data needs medical equipment and it cannot be applied in real time, we proposed an assessment method using eye movement data. Numerous scholars have recently presented assessment methods using eye movement data; however, the environment of their experiment was either natural or traditional displays [29, 31, 32]. When HMDs are utilized, the user’s eyes are remarkably closer to the screen compared with traditional displays. In addition, HMDs imposes more stress to users’ eyes than traditional displays due to their close distance to the screen and immersive environment. No study has investigated individuals’ eye movement when HMD is used due to limitations in eye tracker. In the present study, we supervised the subjects’ eye movement using an eye tracker embedded in the HMD. We extracted 10 eye movement features based on previously conducted studies [30–32]. Jansen et al. [30] defined fixation as an instant when our eyes were stationary and focused on an area interpreting data. It also has been reported that EBR is more sensitive to workload than other conventionally used eye-tracking measures, such as saccade rate and amplitude in a demanding visual task [31]. Changes in blinking habit indicated that a subject may suffer from a high level of eye fatigue. The saccade length was defined as distance (in pixels) between two sequential fixation points in a scanpath [32]. We also ranked the ten eye movement features to assess eye fatigue by using a feature extraction algorithm. Afterward, by using the measurements related to optometry test as the ground truth and the eye movement features as input features, we established ten eye movement feature sets and one blink feature set with different dimensions based on the ranking, and attempted to perform SVM for assessment. Table 5 shows the results of the three kinds of classifications of eye fatigue, including two-class, three-class, and four-class classifications. When comparing the performance of the eleven input feature sets, it was unveiled that the more dimensions of the feature set, the better the result of the classification. The accuracies of the blink set were sightly lower than feature set 10, which were 0.8763, 0.7458, and 0.7225 in the two-class, three-class, and four-class classification, respectively. As presented in Table 6, there were two kinds of regressions of eye fatigue. The input features were as same as multi-class classifications. The accuracies of the blink set were also slightly lower than feature set 10. We also presented two assessment models: eye tracker model and blink detector model. The general preferences and the accuracies of the two models are shown in Table 7. The eye tracker model uses feature set 10 as input data. The blink detector model uses blink set as input data. Both models could provide graded and continuous results to evaluate eye fatigue of HMDs users via analysis of parameters related to eye tracker. Further research should be conducted to verify our findings and explore new practical scenarios.

## Conclusion

In the present study, an objective algorithm to estimate eye fatigue caused by an HMD using eye-tracking data is developed. The experiment had two sessions: the main experiment and the control. In the main experiment, there were four times of SSQ and optometry test, and three times of utilizing HMD, with a total duration of 35 min per participant. The participants’ eyes went from a completely relaxed state to becoming quite fatigued during the experiment. We monitored the participants’ eye conditions during the whole process. SSQ was used to evaluate VIMS. We used a weighted combination of seven indicators of optometry as the ground truth of eye fatigue, which is a novel definition based on our previous study. On the basis of SSQ and optometry data, we found that the relation between VIMS and the feeling of eye fatigue was small. We also demonstrated that subjective perception is not an accurate indicator of eye fatigue. An unobtrusive eye tracker installed in the VR gear was used to perform eye fatigue assessment. Ten eye movement features, including four fixation features, four blink features, and two distance features, were recorded for assessment of eye fatigue. One feature selection algorithms were applied to rank the ten eye movement features based on a particular classifier. We conducted three kinds of classifications and two kinds of regressions to evaluate the performance of different feature sets. We presented two assessment models: eye tracker model and blink detector model. Both models provided graded result and continuous result to users. The models proposed in the present study could be applied to all users.

## Methods

### Overview of experiment

**Fig. 4:**
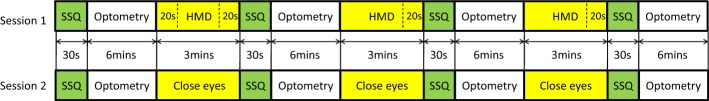
Experiment overview. Session 1 is the main experiment. Session 2 is the control experiment. Each session includes four times of simulator sickness questionnaire (SSQ), four times of optometry test, and three segments of utilizing the HMD

A total of 105 subjects, who aged between 19 and 51 years old, participated in the experiment. All the subjects were with normal or corrected to normal vision. In order to ensure that the initial conditions were the same, all the subjects were fully relaxed before the experiment. Every subject was asked to observe distance (more than 5 m) for more than 30 min before the experiment. With commencement of the experiment, every subject was asked whether his/her eyes were tired or not to make sure he/she was fully relaxed. We assumed that every subject’s eyes experienced no fatigue only before the experiment. Figure 4 shows the experimental procedure. Session 1 presented the main experiment. We monitored the subjects’ eye movement in this session. Session 2 described the control experiment. Every subject participated in these two sessions on different days, while at the same time of the day. Each session included four times of questionnaire, four times of optometry test, and three segments of utilizing HMD. In session 1, subjects watched a video for 3 min in an HMD. An eye tracker was embedded in the HMD, which fully recorded the subjects’ eye movement data. In order to be time efficient, we analyzed the data achieved in four periods of 20 s rather than 3 min. In session 2, subjects still wore the HMD at the same time as session 1, while their eyes were closed during these three parts. Besides, both sessions lasted for 35 min. During filling of the questionnaire, the participants evaluated their feelings on a four-grade quality scale. In the optometry test, we assessed seven parameters on each participant. Optometrists carried out the experiment. The total time spent in this section was 6 min. The same optometrist conducted the whole experiment on the same subject to maintain consistency.

**Fig. 5:**
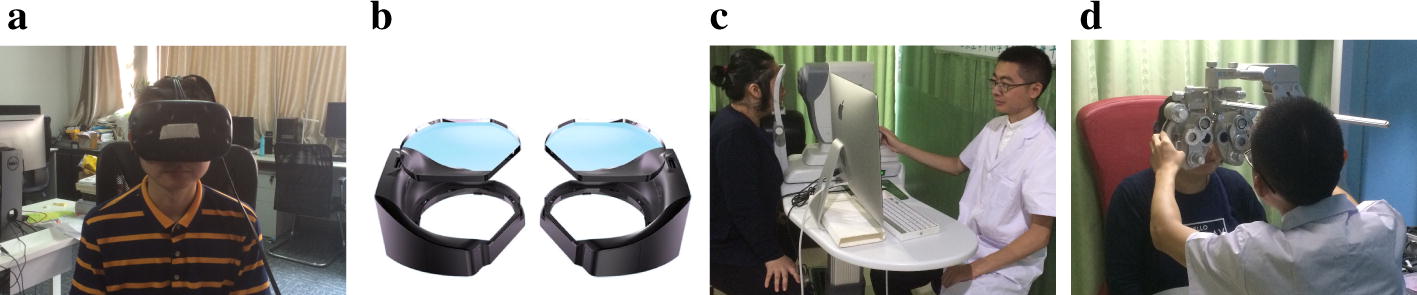
**a** HMD, **b** eye tracker [34], **c** optical biometer, **d** phoropter. Permission to publish photograph was obtained via a signed consent to publish document

As shown in Fig. 5a, the subject wore an HTC Vive HMD in the experiment, consisting of one headset, two controllers, and four base stations. The four base stations were positioned with the headset and controllers in a room with the area of $6.6\times 5$ square meters room. The screen had a resolution of $1080\times 1200$ pixels/eye, and $2160\times 1200$ pixels were combined. The screen’s highest refresh rate was 90 Hz. As illustrated in Fig. 5b, we used aGlASS DKII (Beijing 7invensun Technology Co., Ltd., China) [34] in the experiment. The two black components were manually placed into the headset. Each part could emit infrared light to read the position of each eyeball. The refresh rate was 75 Hz. The error of the tracking position was less than $0.5^\circ$, and the delay was less than 10 ms [48]. We tested the accuracy of the eye tracker before the experiment. We aimed to design a model that could read the value of user’s eye fatigue at any given time during the application of HMD. The system computed eye movement features in real time and imported the data into the assessment model. The lenses shown in Fig. 5b were used to replace the user’s glasses if they wouldn’t like to wear their own. However, since the lenses in aGLASS DKII only had three scales of diopter, which were not appropriate for everyone, participants were asked to wear their own glasses throughout the video-viewing process.

**Table 8:** Items and scoring rule of SSQ

	N	O	D
General discomfort	1	1	
Fatigue		1	
Headache		1	
Eye fatigue		1	
Difficulty focusing		1	1
Increased salivation	1		
Sweating	1		
Nausea	1		1
Difficulty concentrating	1	1	
Fullness of head			1
Blurred vision		1	1
Dizzy (eyes open)			1
Dizzy (eyes closed)			1
Vertigo			1
Stomach awareness	1		
Burping	1		
Total	[1]	[2]	[3]

### SSQ test

We used a traditional questionnaire, the SSQ [25], to assess VIMS. Table 8 presents the items and scoring rule. We utilized a 4-point scale, in which each symptom’s variable score (0,1,2,3) was multiplied by an appropriate weight, and the weighted values were summed down the column to obtain the weighted total. The N, O, and D scores were then calculated from the total values using the conversion formulas given at the bottom of Table 8. The scoring rule is as follows:

1 $$\begin{aligned} score=[1]+[2]+[3]\times 3.74 \end{aligned}$$

### Optometry test

We obtained seven indicators for optometry test, including *BCC*, *PRA*, *NRA*, *PL*, *PR*, *LTL*, and *LTR*. The test was conducted by qualified optometrists. When *BCC*, *PRA*, and *NRA* were tested, the distance between the eye and the target was 40*cm*. The detailed measurement process was described in our previous study [35]. As illustrated in Fig. 5c, d, we herein used two ophthalmic devices: a phoropter and an optical biometer. The phoropter was NIDEK RT-600 (NIDEK Co., Ltd, Japan). It was a comprehensive refractometer, and it was also a favorable device for optometrists to accurately perform optometry. Although an automatic phoropter could provide an acceptable starting point in optometry, it could never replace subjective refraction [49]. We achieved indicators of the three accommodative amplitudes through the phoropter, including ${\text {BCC}}$, ${\text {PRA}}$, and ${\text {NRA}}$, which were discrete and the minimum step size was 0.25 diopter. The optical biometer was SUOER SW-9000 (Suowei Electronic Technology Co., Ltd., China). It provided the indicators of pupil diameters and lens thicknesses for both eyes.

### Definition of eye fatigue

A ground truth function for eye fatigue is given based on our previous study [35] as follows:

2 $$\begin{aligned} F=g({\text{BCC, PRA, NRA, PL, PR, LTL, LTR}}) \end{aligned}$$

where ${\text{BCC}}$, ${\text{PRA}}$, $\mathrm {NRA}$, $\mathrm {PL}$, $\mathrm {PR}$, $\mathrm {LTL}$, and $\mathrm {LTR}$ represent seven indicators for optometry that we previously mentioned in Background section. Besides, *F* is the ground truth function of the eye fatigue. Those seven indicators were simultaneously obtained. In our previous research [35], the ground truth function for individual’s eye fatigue was simply defined as

3 $$\begin{aligned} F=\mathrm {BCC+PRA-NRA-PL-PR-LDL-LDR} \end{aligned}$$

The minuses shown before $\mathrm {NRA}$, $\mathrm {PL}$, $\mathrm {PR}$, $\mathrm {LDL}$, and $\mathrm {LDR}$ indicate that these five indicators were attenuated over time. According to the trends presented in Table 2, we redefined eye fatigue as Eqs. (4) and (5), preventing any single large numerical change due to the fluctuation of all indicators of optometry test.

4 $$\begin{aligned} F=w_1\cdot\text{BCC}+w_2\cdot\text{NRA}+w_3\cdot\text{PRA}+w_4\cdot \text{PR}+w_5\cdot \text{PL}+w_6\cdot \text{LDR}+w_7\cdot \text{LDL} \end{aligned}$$

where $w_i, i=1,2 \ldots 7$ can be formulated in the following equation:

5 $$\begin{aligned} w_i=\frac{1}{{\text{mean}}_{{\rm time}_4}-{\text{mean}}_{{\rm time}_1}}, \quad i=1,2 \ldots 7 \end{aligned}$$

where ${\text{mean}}_{\text{time}_4}$ is the mean value of the corresponding indicator for optometry test in the 4th measurement and ${\text{mean}}_{{\rm time}_1}$ is the mean value of the corresponding indicator of optometry test in the 1st measurement.

### Eye movement test

The distance between the subject and the screen was 2 cm. During the video-watching experience, participants were able to freely blink and move their eyes and heads. In theory, the longer the experiment, the more notable the symptoms of eye fatigue would become. For ethical and humanitarian reasons, the total duration of observation in each subject’s HMD was set to 9 min. We selected ten features related to eye movement, including four features related to fixation (the number of fixation points, the total duration of fixation points, the mean duration of fixation points, and the standard deviation of the duration), four features related to blinking (the times of blinking, the total duration of blinking, the mean duration of blinking, and the standard deviation of the duration), and two features related to scanpath (the length of scan path and the mean length of saccades).

The output of our eye tracker data included users’ gaze locations and their corresponding times. We, in the present study, used Dispersion-Threshold Identification (I-DT) algorithm to obtain the number of fixation and duration of measurement. The minimum duration threshold was set to 200 ms [50]. The dispersion threshold was set to $1^{\circ }$ of visual angle. Blinking features were achieved at the same time of the extraction of fixation features. If the user blinked, the eye tracker was not able to detect the eyes and output data would not include gaze information. Therefore, we can assume that blinking occurred at this time. The duration of the consecutive null data in one time refers to duration of the blink at this time. Although an extreme gaze could also cause this condition, we did not consider this condition. Generally speaking, an extreme gaze happens when the peripheral vision picks up a very strong stimulus at the edge of the display. However, when using the HMD, the user can freely move his/her head, and the screen is always in front of his/her eyes. In the present study, scanpath features were defined by a saccade-fixate-saccade sequence on a display [51]. Here, the scanpath length and the mean length of the saccades were analyzed. The length of saccade was defined as a distance (in pixels) between two sequential fixation points in a scanpath. The scanpath length (in pixels) was taken as summation of the length of saccade in a certain period of time. As shown in Fig. 4, we analyzed eye movement in four periods, in which duration of each period was 20 s. These four periods are the closest periods to the four measurements of optometry test. In order to be time efficient, we analyzed the data obtained during 20 s rather than the 3 min. It lasted for 0.235s to compute all the eye movement features obtained during 20 s for one subject, while that lasted for 0.705s to compute all the features obtained during 1 min.

### Eye fatigue assessment

We proposed an objective assessment for eye fatigue through the use of eye-tracking features. Based on the collected eye-tracking features, the assessment function is given by Eq. (6) as follows:

6 $$\begin{aligned} F=f(\text{NF, DF, MF, VF, BT, DB, MB, VB, SL, ML}) \end{aligned}$$

where $\text{NF}$ is the number of fixation points, $\text{DF}$ represents the total duration of fixation points, $\text{MF}$ denotes the mean duration of fixation points, *VF* denotes standard deviation of the fixation duration, $\text{BT}$ is the times of blinking, $\text{DB}$ represents the total duration of blinking, $\text{MB}$ is the mean duration of the blinking, $\text{VB}$ represents standard deviation of the blinking duration, $\text{SL}$ is the scanpath length, $\text{ML}$ denotes the mean length of the saccades, and *F* is an estimated fatigue value of the user’s eyes at a certain period of time. Equation (6) is a real-time assessment function. All the above-mentioned ten parameters were simultaneously accumulated in a certain period of time. In this study, this period was set to 20 s.

#### Input data

The input data of our assessment model included the ground truth of the eye fatigue and the ten eye movement features. Since all the subjects’ eyes were fully relaxed before the experiment, the ground truth and the ten features needed to be unified. All the 105 subjects were asked to fully relax their eyes before the experiment. We defined the eye fatigue value of this condition equal to 0. To date, for all the subjects, the ground truth values of eye fatigue were all equal to 0 before the 1st measurement using the HMD. In Table 2, however, we noticed that different subjects had different values of the indicators in the 1st optometry test. Thus, we simply unified the ground truth and the features as

7 $$\begin{aligned} \Vert F_{\text{{time}}_i}\Vert =F_{\text{{time}}_i}-F_{\text{{time}}_1} \quad \ i=1,2,3,4 \end{aligned}$$

where $F_{\text{{time}}_i}$ denotes the weighted fatigue value of the *ith* measurement in optometry test, which is computed by Eq. (4). The $\Vert F_{\text{{time}}_i}\Vert$ is the unified ground truth. $\Vert F_{{\text{time}}_{1}}\Vert$ for all the subjects in our experiment was equal to 0. In the same way, the ten eye movement features were unified by Eq. (8) as follows:

8 $$\begin{aligned} \Vert E_{{\text{time}}_i}\Vert =E_{{\text{time}}_i}-E_{{\text{time}}_1} \quad \ i=1,2,3,4 \end{aligned}$$

where $E_{{\text{time}}_i}$ is one of the ten features in Eq. (6) of the *i*th measurement in eye movement test, and $\Vert E_{{\text{time}}_i}\Vert$ is the unified feature.

We then simply discretized eye fatigue by an equal step classification system, and that is given by Eq. (9)

9 $$\begin{aligned} \text{step}=\left( \max _{\Vert P\Vert \in S}[\Vert P\Vert ]-\min _{\Vert P\Vert \in S}[\Vert P\Vert ]\right) /n \end{aligned}$$

where *P* denotes weighted eye fatigue value or one of the eye movement features, *S* includes 420 samples, and *n* represents the number of classes. For instance, if there is a 2-class classification, the $\Vert F\Vert <step$ is set to 0 and the $\Vert F\Vert \ge step$ is set to 1.

#### Feature selection

MRMR criterion is a promising feature selection algorithm [42]. The criterion is a mutual-information-based feature selection. Given two random variables *x* and *y*, their mutual information is defined in terms of their probabilistic density functions *p*(*x*), *p*(*y*) and *p*(*x*, *y*):

10 $$\begin{aligned} I(x;y)=\int \int p(x,y)\text{log}\frac{p(x,y)}{p(x)p(y)}\, \text{d}x\text{d}y \end{aligned}$$

Given a feature set *S* with *m* features ${x_i}$, which jointly have the largest dependency on the target class *c*, the maximal relevance criterion is to search for features, satisfying

11 $$\begin{aligned} \max D(S,c), D=\frac{1}{\mid S \mid }\sum _{x_{i}\in S} I(x_{i};c) \end{aligned}$$

The minimal redundancy criterion is to search for features, satisfying

12 $$\begin{aligned} \min R(S), R=\frac{1}{\mid S \mid ^{2}}\sum _{x_{i},x_{j}\in S} I(x_{i};x_{j}) \end{aligned}$$

The criterion combining these two constraints is called “minimal-redundancy-maximal-relevance”:

13 $$\begin{aligned} \max (D-R) \end{aligned}$$

MRMR has two functions: (i) rank the features; and (ii) reduce the dimension of the features. As we investigated the features with different dimensions in Results section, we only used MRMR to rank the features. However, the published MRMR software package [42] can only accept discrete ground truth data. Thus, we ranked the ten features in three kinds of classifications as follows: two-class classification, three-class classification, and four-class classification.

#### Modeling eye fatigue

The fatigue assessment was undertaken on the basis of SVM. Herein, we conducted three kinds of classifications and two kinds of regressions. The penalty coefficients were set to 1 for all the SVMs. We collected 420 samples from 105 participants for every feature set. We used the holdout method to conduct cross-validation. Eventually, 320 and 100 samples were used for training and testing, respectively. We performed 100 rounds of cross-validation for making a fair comparison. In each round of validation, 100 testing samples were randomly selected.

## Data Availability

The data used and analyzed during the current study are available from the corresponding author on a reasonable request.

## Abbreviations

BCC: binocular crossed cylinder test
CFF: critical fusion frequencies
EGG: electroencephalography
HMD: head-mounted display
LTL: left lens thickness
LTR: right lens thickness
MRMR: minimal-redundancy-maximal-relevance
NRA: negative relative accommodation
PL: left pupil diameter
PR: right pupil diameter
PRA: positive relative accommodation
SSQ: simulator sickness questionnaire
SVM: support vector machine
VIMS: visually induced motion sickness
VR: virtual reality
